# Editorial: Methods and applications in molecular psychiatry: 2023

**DOI:** 10.3389/fpsyt.2024.1536404

**Published:** 2024-12-18

**Authors:** Sehyoun Yoon, Aye-Mu Myint, Karl Bechter

**Affiliations:** ^1^ Feinberg School of Medicine, Northwestern University, Chicago, IL, United States; ^2^ Maastricht University, Maastricht, Netherlands; ^3^ Department of Psychiatry and Psychotherapy II, Bezirkskrankenhaus Günzburg, University of Ulm, Ulm, Germany

**Keywords:** neurodevelopmental disorders, psychiatric disorders, ectodomain shedding, Havana Syndrome, biomarker, human pluripotent cells

The intricate molecular processes governing brain development and function are central to understanding neurodevelopmental and psychiatric disorders. Recent research has emphasized novel methodologies and biomarker discovery to elucidate these mechanisms, advancing our knowledge of disease etiology and potential therapeutic targets. This Research Topic covers findings from studies on ectodomain shedding, Havana Syndrome (HS) bio-behavioral pathways, and D-amino acid biomarkers, alongside insights into human pluripotent stem cell-derived models.

Lobete et al. introduced a protocol for analyzing ectodomain shedding in the central nervous system, emphasizing its role in brain development and disease. By isolating shed ectodomains from mouse brains using ultracentrifugation and mass spectrometry, the authors demonstrated the functional similarities of these proteins to known sheddomes. This approach overcomes the limitations of traditional methods that rely on cell cultures or cerebrospinal fluid samples, offering a more accurate reflection of *in vivo* processes.

Chacko et al. leveraged automated text-mining to identify bio-behavioral pathways implicated in HS, a condition marked by diverse symptoms such as cognitive deficits and vestibular disturbances. Their analysis suggests that the characteristic down or upregulation of almost all predicted HS markers could potentially be caused by pulsed low-frequency electromagnetic fields, including radio frequency radiation. The observed similarities between HS and traumatic brain injury symptoms underscore the need for further research to refine these findings and elucidate potential mechanisms.

Garofalo et al. explored the role of D-amino acids, particularly D-serine (D-Ser) and D-aspartate (D-Asp), as biomarkers for psychiatric disorders like schizophrenia (SCZ) and autism spectrum disorders. Their findings highlight significant reductions in D-Ser and D-Asp levels in SCZ patients, suggesting their relevance for diagnostic and therapeutic applications. However, the study emphasizes the need for larger-scale research to establish reliable cut-off points and assess the biomarkers’ clinical utility in monitoring treatment response.

Finally, Ni et al. reviewed advances in using human pluripotent stem cell-derived cortical interneurons (cINs) to model neurodevelopmental disorders. Their analysis highlights the importance of cINs, particularly those derived from the medial ganglionic eminence, in maintaining excitation-inhibition balance in the brain. Two primary methods for generating cINs—induction via signaling molecules and direct genetic manipulation—offer distinct advantages and limitations. The article provides examples of how these methods are applied to study disease mechanisms and addresses challenges such as achieving full neuronal maturity.

Overall, this Research Topic emphasizes the transformative potential of molecular approaches in understanding and addressing neurodevelopmental and psychiatric disorders. By refining methodologies, uncovering novel biomarkers, and advancing disease models, these studies pave the way for personalized interventions and enhanced patient outcomes.

